# Health-Related Quality of Life After Neonatal Treatment of Symptomatic Tetralogy of Fallot: Insights from the Congenital Cardiac Research Collaborative

**DOI:** 10.1007/s00246-024-03650-2

**Published:** 2024-09-21

**Authors:** George T. Nicholson, Jeffrey D. Zampi, Andrew C. Glatz, Bryan H. Goldstein, Christopher J. Petit, Yun Zhang, Courtney E. McCracken, Athar M. Qureshi, Caren S. Goldberg, Jennifer C. Romano, Mark A. Law, Jeffery J. Meadows, Shabana Shahanavaz, Sarosh P. Batlivala, Shiraz A. Maskatia, Asaad Beshish, Michael L. O’Byrne, R. Allen Ligon, Kathryn O. Stack, Hala Q. Khan, Shalin Parekh, Dawn L. Ilardi

**Affiliations:** 1 Division of Cardiology, Department of Pediatrics, Vanderbilt University School of Medicine, Nashville, TN USA; 2 Division of Cardiology, Department of Pediatrics, C.S. Mott Children’s Hospital, University of Michigan School of Medicine, Ann Arbor, MI USA; 3 Division of Cardiology, Department of Pediatrics, Washington University School of Medicine, St. Louis, MA USA; 4 Department of Pediatrics, Heart Institute, UPMC Children’s Hospital of Pittsburgh, University of Pittsburgh School of Medicine, Pittsburgh, PA USA; 5 Morgan Stanley Children’s Hospital, Columbia University Vagelos College of Physicians & Surgeons, New York, NY USA; 6 Center for Research and Evaluation, Kaiser Permanente Georgia, Atlanta, GA USA; 7 Lillie Frank Abercrombie Division of Cardiology, Department of Pediatrics, Texas Children’s Hospital, Baylor College of Medicine, Houston, TX USA; 8 Section of Pediatric Cardiothoracic Surgery, Department of Cardiac Surgery, C.S. Mott Children’s Hospital, University of Michigan School of Medicine, Ann Arbor, MI USA; 9 Division of Pediatric Cardiology, Department of Pediatrics, Children’s of Alabama, University of Alabama Birmingham School of Medicine, Birmingham, AL USA; 10 Division of Cardiology, Department of Pediatrics, University of California San Francisco School of Medicine, San Francisco, CA USA; 11 The Heart Institute, Cincinnati Children’s Hospital Medical Center and Division of Pediatric Cardiology, University of Cincinnati College of Medicine, Cincinnati, OH USA; 12 Division of Pediatric Cardiology, Department of Pediatrics, Stanford University School of Medicine, Stanford, CA USA; 13 Children’s Heart Center Cardiology, Department of Pediatrics, Children’s Healthcare of Atlanta, Emory University School of Medicine, Atlanta, GA USA; 14 The Cardiac Center at the Children’s Hospital of Philadelphia, Philadelphia, PA USA; 15 Connecticut Children’s Hospital, Hartford, CT USA; 16 Pediatric Neurodevelopmental Center, Atlanta, GA USA; 17 Thomas P. Graham Jr. Division of Pediatric Cardiology, Pediatric Heart Institute, Vanderbilt University Medical Center, 2200 Children’s Way, 5230 Doctors’ Office Tower, Nashville, TN 37232 USA

**Keywords:** Congenital heart disease, Tetralogy of fallot, Quality of life

## Abstract

To evaluate the association between initial management strategy of neonatal symptomatic Tetralogy of Fallot (sTOF) and later health-related quality of life (HRQOL) outcomes. We performed a multicenter, cross-sectional evaluation of a previously assembled cohort of infants with sTOF who underwent initial intervention at ≤ 30 days of age, between 2005 and 2017. Eligible patients’ parents/guardians completed an age-appropriate Pediatric Quality of Life Inventory, a Pediatric Quality of Life Inventory Cardiac Module Heart Disease Symptoms Scale, and a parental survey. The association between treatment strategy and HRQOL was evaluated, and the entire sTOF cohort was compared to published values for the healthy pediatric population and to children with complex congenital heart disease and other chronic illness. The study cohort included 143 sTOF subjects, of which 59 underwent a primary repair, and 84 had a staged repair approach. There was no association between initial management strategy and lower HRQOL. For the entire cohort, in general, individual domain scores decreased as age sequentially increased. Across domain measurements, mean scores for the sTOF cohort were significantly lower than the healthy pediatric population and comparable to those with other forms of complex CHD and other chronic health conditions. The presence of a genetic syndrome was significantly associated with a poor HRQOL (*p* = 0.003). Initial treatment strategy for sTOF was not associated with differences in late HRQOL outcomes, though the overall HRQOL in this sTOF cohort was significantly lower than the general population, and comparable to others with chronic illness.

**Supplementary Information:**

The online version contains supplementary material available at 10.1007/s00246-024-03650-2.

## Introduction

Advances in healthcare have resulted in significant improvement in the survival of pediatric patients with chronic medical conditions, including infants with complex congenital heart disease, CHD [1]. Tetralogy of Fallot (TOF) is the most common form of cyanotic CHD [2]. A subset of neonates with TOF are symptomatic (sTOF) due to ductal dependence or the severity of cyanosis, necessitating early intervention. Management strategies for sTOF neonates include primary repair or staged repair, which consists of an initial palliation followed by later complete repair. Previously, the Congenital Cardiac Research Collaborative (CCRC) performed a large, multicenter study of neonates with sTOF; this study demonstrated an early survival benefit with staged repair, but this difference was mitigated over medium-term follow-up [3].

Since the overall survival of infants with CHD has significantly improved, the assessment of long-term functional outcomes associated with survival is paramount [4]. Neurodevelopmental outcome research in TOF highlights vulnerabilities across multiple domains, including intellectual functioning, language, visuospatial processing, and executive functioning [5–9]. Higher rates of anxiety, attention-deficit-hyperactivity disorder (ADHD), and autism are also concerning [6, 10]. Disease severity and associated genetic disorders lead to an increased risk of neurodevelopmental challenges in children with TOF [11]. Given the resources and time required for neurodevelopmental evaluation and early intervention, research to date shows low rates of return for children with CHD generally [12].

Health-related quality of life (HRQOL) measures are an efficient and effective way to screen for the impact of a medical condition on an individual’s everyday life in the areas of physical, emotional, social, and academic functioning [13]. These standardized rating scales are completed by the patient and/or a proxy (usually their parent/guardian); thus, these tools offer valuable information about beliefs, perceptions, and discrepancies between respondents [14]. As infants with CHD require lifelong follow-up, HRQOL measures are also a component of quality, comprehensive care to help determine the value of healthcare services [15, 16]. This is particularly germane in the TOF population, as repeated medical interventions are often required and could lead to changes in clinical outcomes. Yet, despite numerous studies evaluating outcomes of patients with TOF, relatively few have evaluated HRQOL, and even fewer compare HRQOL to clinical outcomes [17–20]. In addition, it remains unclear how HRQOL may change over time throughout childhood. In our population of interest, sTOF, although there was no difference in mortality between treatment strategies, the overall morbidity burden favored those who underwent a primary repair [3].

The purpose of the current study was to assess the association between initial neonatal management strategy and HRQOL outcomes at different stages of development. Further, we sought to explore parent- and patient-specific factors associated with lower HRQOL following neonatal intervention for sTOF. Given the differences in exposures associated with the range of neonatal sTOF treatment strategies, we hypothesized that sTOF initial treatment pathway will result in differences in HRQOL scores later in life.

## Methods

A multicenter, cross-sectional evaluation of a previously assembled cohort included all infants with sTOF who underwent initial intervention at ≤ 30 days of age from January 1, 2005, through November 30, 2017 at nine participating centers in the CCRC. The index procedure consisted of either primary repair or initial palliation. The results of the main study analysis have been previously published [3]. All living subjects included in the prior analysis were eligible for inclusion in this study. Data collection was performed by individual centers under the direction of the site principal investigators and rigorous electronic data auditing was performed [21]. Data with a limited set of identifiers were then aggregated and analyzed at Children’s Healthcare of Atlanta, which served as the data coordinating center for the CCRC. This study was approved by the Institutional Review Board at Cincinnati Children’s Hospital, which acted as the single Institutional Review Board, with a waiver of the need for informed consent. A data-use agreement was in place among all participating centers and the data coordinating center. Sharing of patient-level data is prohibited by the terms of these data-use agreements. Statistical methods and code will be shared upon request.

Subject eligibility was determined by medical record review, as all patients who were not known to be deceased were eligible for inclusion. The eligible patient’s parents/guardians were mailed a research packet which included an introductory letter, age-appropriate Pediatric Quality of Life Inventory (PedsQL™), Pediatric Quality of Life Inventory Cardiac Module Heart Disease Symptoms Scale (PedsQL™ CM), and a parental survey. For further information regarding the PedsQL™ scale, one can visit https://www.pedsql.org. No family was contacted directly to participate in the study. Return of the postage-paid research materials was an inclusion criterion and an indication of implied consent. Exclusion criteria included a failure to return research materials or return of incomplete material. If, after the initial mailing, study materials were not returned, a second research packet was resent to the parents/guardians 3–6 months later. No further contact was attempted if study materials were not returned after the second mailing. Completed research packets were returned to individual study sites and de-identified locally. The de-identified study documents were electronically transmitted to the data coordinating center for entry, scoring, and evaluation.

PedsQL™ version 4.0 is a 23-item questionnaire encompassing Physical, Emotional, Social, and School Functioning [22]. The total scale score measures overall generic HRQOL, with a higher score indicating better HRQOL. The PedsQL™ CM version 3.0 comprises items and domains that apply to children with heart disease [23]. This includes the following cardiac-specific scales: Heart Problems and Treatment, Treatment II, Perceived Physical Appearance, Treatment Anxiety, Cognitive Problems, and Communication. The mean scale scores are calculated as the sum of the items divided by the number of items answered. Items are reverse scored and linearly transformed, meaning that lower scores indicate more heart disease problems resulting in a lower HRQOL.

A Parent Survey Questionnaire was created for the current study to collect information about child and parent demographics (e.g., parent education, language spoken at home), development and health status (e.g., feeding, developmental diagnoses), and access to educational and intervention resources (e.g., school plan, speech therapy, etc.), all of which may impact HRQOL (Supplemental Table).

### Statistical Analyses

Demographic and clinical characteristics were compared between respondents and non-respondents, with counts and percentages reported for categorical variables and medians (25th–75th percentiles) for skewed continuous data. Skewed continuous data were compared using Wilcoxon rank sum tests. Categorical variable comparisons were made using *χ*^2^ tests, and Fisher’s exact test was used when expected cell counts were less than 5.

Using Wilcoxon rank sum tests, the results of our sTOF cohort were compared to previously published values from a population of healthy children, children with chronic health conditions, and children with other forms of complex CHD [24]. To evaluate our primary aim which was to determine the association of treatment strategy with HRQOL, we compared those patients who underwent a staged repair approach versus primary repair.

We also aimed to explore the association between demographic and clinical characteristics with a poor HRQOL score (defined as a score ≥ 2 standard deviations below the mean for the healthy pediatric population for either the PedsQL™ total score, psychosocial score, or physical score). Continuous variables were compared with patients with normal and poor HRQOL scaled scores using Wilcoxon rank sum tests. Categorical variables were compared using *χ*^2^ tests.

The parental/guardian survey results were summarized, including the level of parental education, receipt of services, additional developmental delay diagnosis(es), and parental concerns with development. Additionally, the survey responses were compared based on normal versus poor PedsQL™ scores using Wilcoxon rank sum test and *χ*^2^ tests. All analyses were conducted using SAS v.9.4 (SAS Institute, Inc., Cary, NC) and statistical significance was assessed at the 0.05 level. An important issue with HRQOL and other multi-domain composite scores is the risk that erroneous associations are found because of multiple comparisons. We mitigated this by identifying our primary outcome(s) during the design of the study. All other analyses are exploratory. No additional steps were taken to mitigate multiple comparisons.

## Results

Of the 572 patients in the primary study, PedsQL™, PedsQL™ CM, and parental surveys were sent to 511 patients expected to be alive at the time of this study. One-hundred and forty-three surveys were returned for a response rate of 28%; the study cohort comprised 60% males with a median respondent age of 8 years (range of 3 to 16 years). Eighteen percent of respondents were born premature, 25% had a diagnosed genetic syndrome, and 26% had an extracardiac anomaly. Within the study cohort, the median age at the index operation was 7 days (IQR 4–14 days), with 59% having undergone a staged repair approach. Across the first 18 months of life, the total duration of cardiopulmonary bypass, inhalational anesthetic, mechanical ventilation, and hospital length of stay are outlined in Table 1. In terms of our primary aim, we did not discover a significant difference in measures of HRQOL between primary versus staged repair strategies (*p* = 0.26).

**Table 1:** Comparison of respondents and non-respondents

Characteristic	Respondent (*N* = 143)		Non-respondent (*N* = 373)		*p*-value
	*N*	%	*N*	%	
Sex (male)	86	60%	194	52%	0.10
Birth weight (kg)	140	2.8 (2.5, 3.3)	364	2.9 (2.5, 3.3)	0.22
Gestational age (weeks)	137	38 (37, 39)	358	38 (37, 39)	0.09
Prematurity (< 37 weeks)	26	18%	71	19%	0.82
Extra cardiac anomaly	37	26%	108	29%	0.49
Any genetic syndrome	36	25%	109	2%	0.53
DiGeorge syndrome	12	8%	41	11%	0.38
Age at time of index operation	143	7 (4, 14)	373	7 (5, 15)	0.55
Inotropic support prior to index procedure	16	11%	40	11%	0.92
Invasive mechanical ventilation prior to index procedure	26	18%	87	23%	0.30
Anatomic diagnosis					
TOF/PS	75	53%	188	50%	0.68
TOF/PA	68	48%	185	50%	
Treatment strategy					
Primary repair	59	41%	153	41%	0.96
Staged repair	84	59%	220	59%	
Duration of bypass (min)*	140	123 (92, 171)	364	130 (89, 186)	0.40
Duration of inhalational anesthetic (min)*	137	354 (225, 495)	331	330 (195, 529)	0.38
Duration of mechanical ventilation (days)*	140	3.0 (1.5, 6.0)	365	4.0 (2.0, 7.0)	0.12
Total ICU LOS (days)*	140	11 (7, 18)	358	10 (6, 18)	0.80
Total hospital LOS (days)*	140	27 (17, 36)	364	26 (16, 41)	0.67
Number of reinterventions*	84	2.0 (1.0, 4.0)	208	2.0 (1.0, 3.0)	0.02

Values reported as *N* (%) or median (25th–75th percentiles)

*ICU* intensive care unit, *LOS* length of stay, *PA* pulmonary atresia, *PS* pulmonary stenosis, *TOF* Tetralogy of Fallot

*Value provided is the total patient value over the first 18 months of life

### Parent-/Guardian-Reported HRQOL

**Fig. 1:**
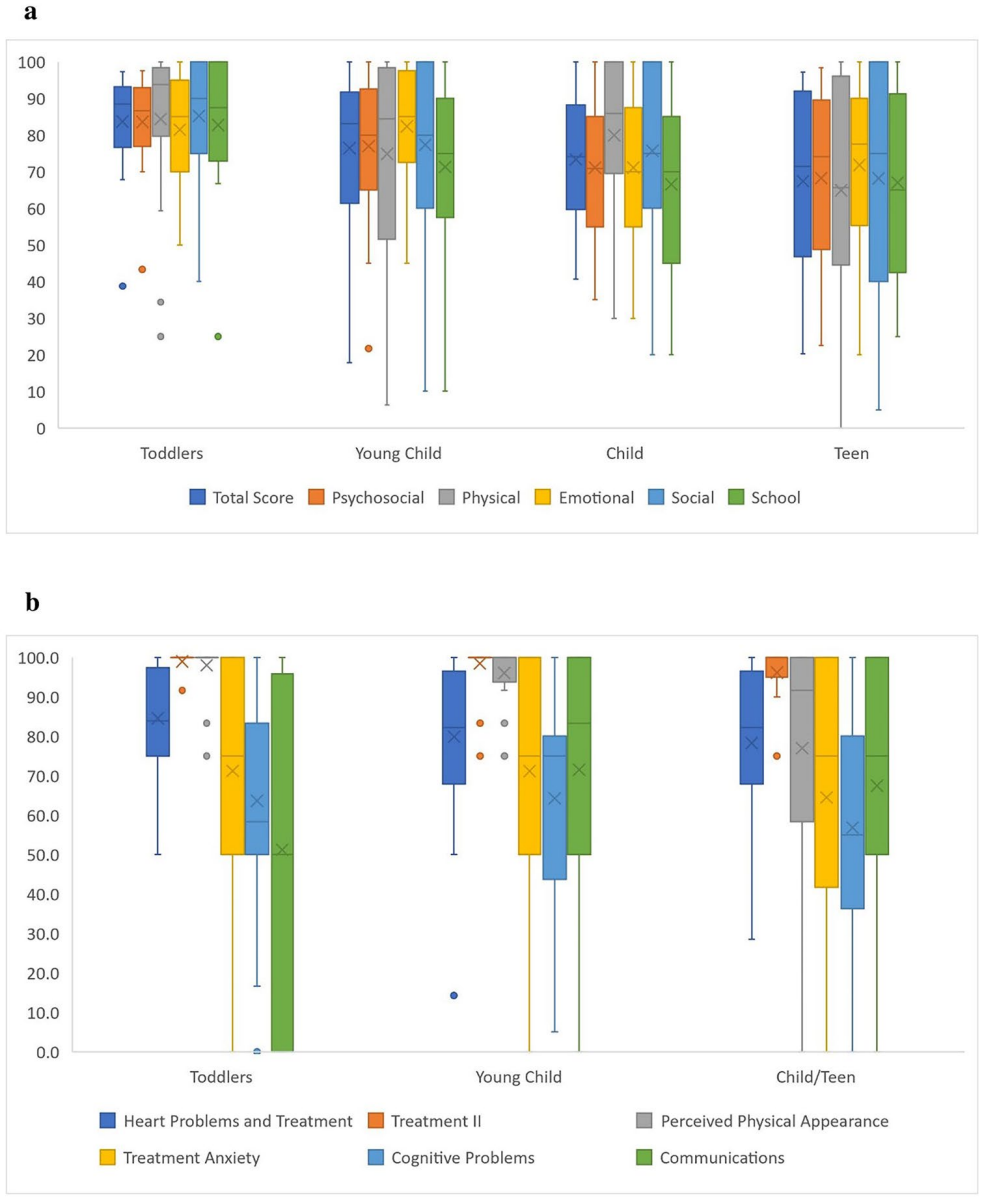
**a** Mean PedsQL™ domain scores by age range. Mean value with standard deviation range displayed. **b** Mean PedsQL™ cardiac module domain scores by age range. Mean value with standard deviation range displayed

The mean total score for the parent-/guardian-reported PedsQL™ was 75.1 (SD 18.7) for the entire cohort. In general, the mean reported score for each domain in the PedsQL™ as well as the total score decreased as the age range sequentially increased from toddlers to teens in this cross-sectional sample. The same phenomenon was observed within each measured domain in the PedsQL™ CM, with the exception of Communication (Tables 2, 3; Fig. 1a, b).

**Table 2:** Quality of life scores for PedsQL™ by age and domain

Age range	Domain	Mean	Standard deviation
Toddlers (2–4 years) *N* = 21	Total score	84	13
	Physical	84	21
	Emotional	81	15
	Social	85	15
	Psychosocial	84	12
	School	83	20
Young Child (5–7 years) *N* = 49	Total score	76	19
	Physical	75	27
	Emotional	82	16
	Social	77	23
	Psychosocial	77	17
	School	71	23
Child (8–12 years) *N* = 52	Total score	73	17
	Physical	80	20
	Emotional	71	20
	Social	76	22
	Psychosocial	71	18
	School	67	23
Teen (13–17 years) *N* = 20	Total score	67	25
	Physical	65	34
	Emotional	72	22
	Social	68	32
	Psychosocial	68	23
	School	67	24

**Table 3:** Quality of life scores for PedsQL™ cardiac module by age and domain

Age range	Domain	Mean	Standard deviation
Toddlers (2–4 years) *N* = 21	Heart Problems and Treatment	85	14
	Treatment II	99	3
	Perceived Physical Appearance	98	6
	Treatment Anxiety	71	27
	Cognitive Problems	64	25
	Communications	51	40
Young Child (5–7 years) *N* = 48	Heart Problems and Treatment	80	20
	Treatment II	99	6
	Perceived Physical Appearance	96	8
	Treatment Anxiety	71	32
	Cognitive Problems	64	25
	Communications	72	33
Child and Teen (8–17 years) *N* = 72	Heart Problems and Treatment	78	20
	Treatment II	96	8
	Perceived Physical Appearance	77	28
	Treatment Anxiety	65	35
	Cognitive Problems	57	27
	Communications	68	31

### HRQOL as Compared to Other Populations

**Fig. 2:**
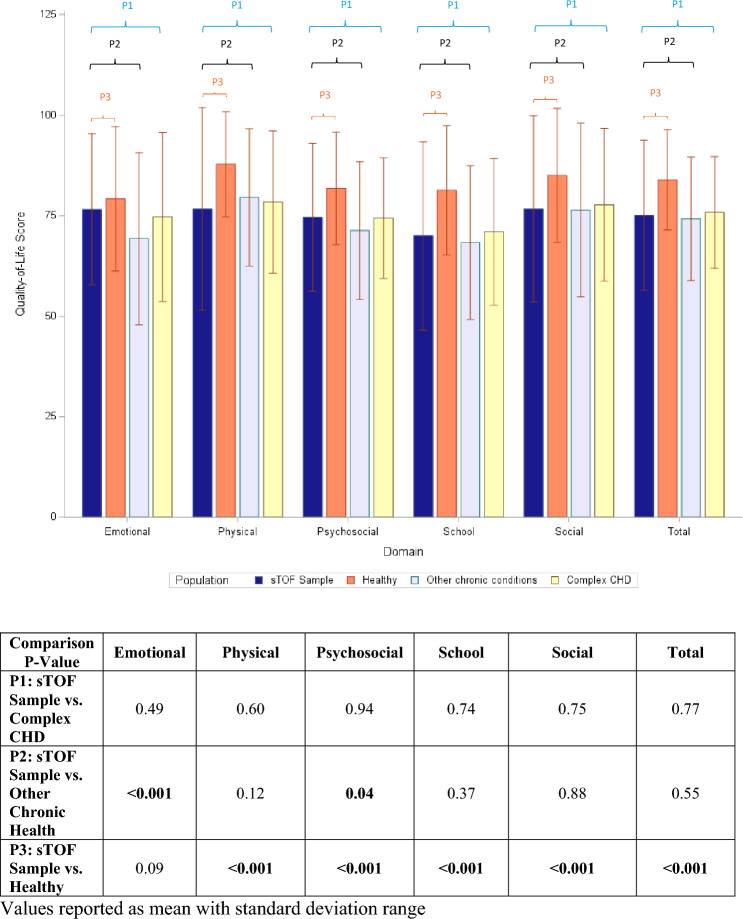
Comparison of Quality-of-Life Scores to Healthy Children and Children with other Chronic Health Conditions

As shown in Fig. 2, the mean total score for the entire cohort was significantly lower than published data for the healthy pediatric population (75 ± 18.7 vs. 84 ± 12.5, *p* < 0.001). When compared to the healthy pediatric population, the sTOF cohort scored significantly lower across all PedsQL™ domains except for Emotional domain (*p* = 0.09). In contrast, when compared to published data on children with chronic health conditions, the sTOF cohort scores were similar except for the Emotional and Psychosocial domains, where the sTOF cohort scored higher (*p* < 0.001 and 0.04, respectively). Additionally, there were no differences across all PedsQL™ domains when comparing this cohort to published data on children with other forms of complex CHD.

Twenty-seven percent of the study cohort had at least one PedsQL™ domain score greater than 2 standard deviations below the mean of the healthy pediatric population. Table 4 specifies the proportion of patients within each domain with significantly low mean scores as compared to the healthy population.

**Table 4:** Children with poor quality of life

Domain	Proportion with worse quality of life*
Total Score	33 (23%)
Physical	38 (27%)
Emotional	6 (4%)
Social	25 (18%)
Psychosocial	25 (18%)
School	29 (21%)

Values reported as number (percentage)

*Poor quality of life is defined as a PedsQL domain score greater than 2 standard deviations below the mean of the healthy pediatric population

### Medical Risk Factors Associated with a Poor HRQOL

As outlined in Table 5, potential influences which may impact HRQOL were selected to investigate potential risk factors associated with a poor HRQOL. The presence of a genetic syndrome was significantly associated with a worse HRQOL (*p* = 0.003). Potentially adverse exposures, such as duration of cardiopulmonary bypass, number of reinterventions, and hospital length of stay, were equivalent between those with normal and poor HRQOL scores.

**Table 5:** Risk factors associated with a poor quality of life

Characteristic	Normal QOL (*N* = 96)	Poor QOL (*N* = 47)	*p* value
Male sex	58 (60%)	27 (59%)	0.85
Birth weight (kg)	2.9 (2.5, 3.3)	2.7 (2.4, 3.1)	0.13
Gestational age (weeks)	38 (37, 39)	38 (37, 39)	0.53
Prematurity (< 37 weeks)	17 (18%)	9 (20%)	0.79
Extra cardiac anomaly	23 (24%)	13 (28%)	0.58
Any genetic syndrome	14 (15%)	17 (37%)	0.003
DiGeorge syndrome	5 (5%)	6 (13%)	0.18
Age at time of index operation (days)	7 (4, 15)	8 (4, 12)	0.84
Inotropic support prior to index operation	11 (12%)	5 (11%)	0.35
Intubated prior to index operation	15 (16%)	11 (24%)	0.23
Anatomic diagnosis			
TOF/PS	52 (54%)	23 (50%)	0.68
TOF/PA	44 (46%)	23 (50%)	
Treatment strategy			
Primary repair	43 (45%)	16 (35%)	0.26
Staged repair	53 (55%)	30 (65%)	
Duration of cardiopulmonary bypass (min)*	121 (93, 171)	122 (88, 162)	0.68
Duration of inhalational anesthetic (min)*	345 (210, 480)	400 (255, 525)	0.21
Number of reinterventions*	2 (1,3)	1 (1,2)	0.13
Hospital complications (yes)	35 (37%)	17 (39%)	0.84
Duration of mechanical ventilation (days)*	3 (2, 5)	3 (1, 7)	0.66
ICU LOS (days)*	10 (6, 15)	13 (7, 21)	0.07
Hospital LOS (days)*	25 (17, 34)	27 (21, 40)	0.15

Poor quality of life defined as a PedsQL™ total, psychosocial, or physical domain score of ≥ 2 standard deviations below the mean

Values reported as *N* (%) or median (25th–75th percentiles)

*Value provided is the total patient value over the first 18 months of life

*ICU* intensive care unit, *LOS* length of stay, *PA* pulmonary atresia, *PS* pulmonary stenosis, *TOF* Tetralogy of Fallot

### Parent/Guardian Survey Responses and HRQOL

Supplemental Table summarizes the parental survey responses. Over 90% of the surveys were completed by a patient’s mother and 92% of the cohort primarily spoke English at home. Approximately, 48% of the mothers and 38% of the fathers had graduated from a 4-year college or obtained a professional degree. Most of the study population were fed completely by mouth (94%) and not requiring supplemental oxygen (97%). Approximately, 61% of the cohort were not receiving any support services. Most of the cohort was receiving additional educational services, either individual educational programs (IEPs), early services or special education. In fact, only 42% of our sTOF cohort were not receiving any educational services. Thirty-one percent of the subjects were diagnosed with a developmental delay, while 33% had a speech delay and 35% had a learning disability. Fifteen percent of the cohort had an autism spectrum disorder.

Only 1.4% of respondents rated their child’s quality of life as fair and 6% rated their child’s overall health as fair. No respondent rated their child’s quality of life or overall health as poor. When respondents were asked if there were any parental concerns across a variety of developmental areas—growth and nutrition, school/learning/development, general health and quality of life, social development, or cardiac concerns—two-thirds of respondents indicated a concern in at least one of the domains.

There were several survey responses that were significantly associated with a poor HRQOL as displayed in Table 6. A poor HRQOL was more likely if a child was receiving a form of therapy or an educational service. Those with developmental delay, a learning disability, autism spectrum disorder, or a sensory deficit disorder were also more likely to be associated with a poor HRQOL. Those with a poor HRQOL were more likely to have a parent/guardian express concern across the entire group of previously mentioned developmental areas.

**Table 6:** Associations of parental survey responses and quality of life

Survey response	Normal QOL *N* = 96	Poor QOL *N* = 47	*p* value
Any parent education ≥ college degree	59 (62%)	22 (47%)	0.10
Currently receiving therapy*	29 (30%)	25 (53%)	0.008
Currently receiving educational services^¥^	44 (46%)	32 (68%)	0.01
Additional diagnosis			
Developmental delay	20 (21%)	22 (47%)	0.001
Speech delay	28 (29%)	19 (40%)	0.20
Vision problems	10 (10%)	10 (21%)	0.08
Motor delay	21 (22%)	16 (34%)	0.20
Sensory skill deficit	6 (6%)	10 (21%)	0.007
Learning disability	6 (6%)	14 (30%)	< 0.001
Autism spectrum disorder	2 (2%)	6 (13%)	0.02
Present concerns			
Growth and nutrition	18 (19%)	17 (36%)	0.02
School, learning, development	29 (30%)	25 (53%)	0.008
Social development	18 (19%)	16 (34%)	0.04
Cardiac concerns	20 (21%)	17 (36%)	0.05
General health and QOL	6 (6%)	9 (19%)	0.04

Poor quality of life is defined as a PedsQL total, psychosocial, or physical domain score of ≥ 2 standard deviations below the mean

Values reported as *N* (%)

*Defined as the individual currently receiving at least one of the following—physical therapy, occupational therapy, speech therapy, or behavioral therapy

^¥^Defined as the individual currently receiving at least one of the following—early education service, IEP/504 plan, or special education

## Discussion

In this multicenter report evaluating the current HRQOL in children and adolescents with sTOF, results reveal a significantly lower HRQOL as compared to previously published normative data for the healthy pediatric population. We found no difference in HRQOL based on initial sTOF management strategy despite a favorable morbidity profile in those that underwent primary repair. The current sTOF sample reported an overall similar HRQOL to prior studies focused on pediatric populations with complex CHD and other chronic health conditions. Notably, we found a very high prevalence of need in children and adolescents following sTOF repair. Neurodevelopmental therapies were necessary in a high proportion of this population early in childhood, while individual school programs and assistance were required in a preponderance of the older cohort. In an additional analysis of potential risk factors associated with a poor HRQOL, only the presence of a genetic syndrome was found to be associated with poorer HRQOL. All other studied clinical or medical risk factors did not relate to HRQOL in this sTOF cohort.

This multicenter, cross-sectional evaluation of repaired sTOF patients is not only the largest pediatric TOF population examining HRQOL to date, but it also expands beyond prior adolescent studies to include younger children. Another unique attribute to our study is the focus on patients with symptomatic TOF necessitating initial intervention in the neonatal period (< 30 days of age). Fetal and neonatal hypoxia are among the mechanisms that contribute to both delayed brain maturation as well as white matter injury in neonates with complex CHD [25–27]. These neurological consequences are then associated with later neurodevelopmental concerns [27–30]. Interestingly, hypercyanotic spells in infancy have been correlated with lower psychosocial outcomes later in life [31].

Our results indicate that HRQOL in school-age children with sTOF is related to parent-reported developmental delays, neurodevelopmental disorders (e.g., autism, learning disability), and need for educational and rehabilitation services. This is similar to previously published reports documenting a lower HRQOL in the presence of a comorbid genetic disorder and worse neurocognitive skills [19, 20, 32]. However, those reports also found an association between greater medical/surgical risks and poor HRQOL, whereas we did not find an association with early clinical exposures and late HRQOL. This difference may be due to the relatively homogenous sample of TOF patients in our cohort as opposed to these previously published studies. It is also important to note that our results reveal the significant need for on-going support regarding therapies, educational services, and medical needs this population faces.

Examination of cardiac-specific HRQOL concerns revealed that anxiety around treatment and cognitive problems were highest. These findings, along with the remarkably high frequency of autism spectrum disorder in our cohort, support the value of monitoring neurodevelopmental and psychosocial concerns in those with high-risk CHD, consistent with the updated scientific statement from the American Heart Association [33], and this could certainly include HRQOL measures.

As HRQOL is a dynamic measure and varies with age, so do the primary drivers which determine overall QOL [34]. Not only do psychosocial factors drive HRQOL in the TOF population [19, 20], present literature also supports the key role of physical functioning in this population [32, 35]. Our present study supports this notion as demonstrated by the dramatic decline in the physical functioning domain from toddlerhood through adolescence, which is a major determinant of HRQOL in childhood. Adolescence is a major transitional life stage, as one attempts to establish independence with a different set of developmental tasks [36]. This period is particularly difficult for those with CHD, in part because physical limitations may become more pronounced [31]. However, in the TOF population, it is unclear if these poor physical domain scores are due to true physical limitations, restrictions imposed by medical providers, or perceived ill health [37].

There are several limitations to the present study. The retrospective nature of this study allows for the possibility of unmeasured confounders that impact HRQOL. The cross-sectional design limits our ability to establish the causation of outcomes and our ability to examine changes in HRQOL over time in the same patient. As we only had a 28% response rate, unmeasured differences between the responders and nonresponders in this study may have resulted in selection bias. An additional confounder was that this study was conducted during the COVID-19 pandemic, which resulted in significant psychological distress [38]. Longitudinal patient-specific studies are needed to determine potential mediators of HRQOL outcomes. Germane to the sTOF population, this would include determination of cardiac arrhythmia and on-going disease burden. Although self-report is considered the standard for measuring perceived HRQOL, the PedsQL™ and PedsQL™ CM utilize parental or proxy report. Although it can be argued that parent-perception of their child’s HRQOL has as a significant influence on health care utilization, a proxy rater’s estimate may be incongruent with that of a patient’s self-report [32].

As recommended by leading scientific organizations, such as the American Heart Association, there is a need for targeted surveillance, identification, and intervention to provide effective and efficient therapies to improve behavioral, psychosocial, and academic performance throughout childhood and into adulthood [33, 39]. This study reinforces the value of screening quality of life in high-risk children with CHD as one strategy for identifying needs and referring for additional clinical management. The discrepancy between parental reports of child well-being (i.e., similar to that which may be shared during clinic visits with a cardiologist) and parent responses on a standardized measure of HRQOL is significant. Our results suggest a difference in the parental perception and objective measures of HRQOL, as only 1.4% of parents responded as rating their child’s HRQOL as “fair” or less, while objective measurement revealed that approximately 25% of the cohort had significantly poor HRQOL. Future studies will need to continue exploring ways to increase the feasibility of how medical teams can screen, monitor, and manage the complex and changing lifespan needs of those with high-risk CHD, including sTOF.

## Conclusion

Although the initial treatment strategy for sTOF and early in-hospital outcomes were not associated with later HRQOL, the overall HRQOL in our cohort was significantly lower than the healthy population, and comparable to others with chronic illness. A lower HRQOL was associated with neurodevelopmental delays, the presence of genetic disorders, and higher need for services.

## Supplementary Information

Below is the link to the electronic supplementary material.

Supplementary file1 (PDF 93 KB)

## Data Availability

Sharing of patient-level data is prohibited by the terms of these data-use agreements. Statistical methods and code will be shared upon request.

## Abbreviations

CCRC: Congenital Cardiac Research Collaborative
CHD: Congenital Heart Disease
HRQOL: Health-Related Quality of Life
PedsQL™: Pediatric Quality of Life Inventory
PedsQL™ CM: Pediatric Quality of Life Inventory Cardiac Module Heart Disease Symptoms Scale
sTOF: Symptomatic Tetralogy of Fallot
TOF: Tetralogy of Fallot
