# Correction: Health-Related Quality of Life After Neonatal Treatment of Symptomatic Tetralogy of Fallot: Insights from the Congenital Cardiac Research Collaborative

**DOI:** 10.1007/s00246-024-03680-w

**Published:** 2024-10-21

**Authors:** George T. Nicholson, Jeffrey D. Zampi, Andrew C. Glatz, Bryan H. Goldstein, Christopher J. Petit, Yun Zhang, Courtney E. McCracken, Athar M. Qureshi, Caren S. Goldberg, Jennifer C. Romano, Mark A. Law, Jeffery J. Meadows, Shabana Shahanavaz, Sarosh P. Batlivala, Shiraz A. Maskatia, Asaad Beshish, Michael L. O’Byrne, R. Allen Ligon, Kathryn O. Stack, Hala Q. Khan, Shalin Parekh, Dawn L. Ilardi

**Affiliations:** 1 Division of Cardiology, Department of Pediatrics, Vanderbilt University School of Medicine, Nashville, TN USA; 2 Division of Cardiology, Department of Pediatrics, C.S. Mott Children’s Hospital, University of Michigan School of Medicine, Ann Arbor, MI USA; 3 Division of Cardiology, Department of Pediatrics, Washington University School of Medicine, St. Louis, MA USA; 4 Department of Pediatrics, Heart Institute, UPMC Children’s Hospital of Pittsburgh, University of Pittsburgh School of Medicine, Pittsburgh, PA USA; 5 Morgan Stanley Children’s Hospital, Columbia University Vagelos College of Physicians & Surgeons, New York, NY USA; 6 Center for Research and Evaluation, Kaiser Permanente Georgia, Atlanta, USA; 7 Lillie Frank Abercrombie Division of Cardiology, Department of Pediatrics, Texas Children’s Hospital, Baylor College of Medicine, Houston, TX USA; 8 Section of Pediatric Cardiothoracic Surgery, Department of Cardiac Surgery, C.S. Mott Children’s Hospital, University of Michigan School of Medicine, Ann Arbor, MI USA; 9 Division of Pediatric Cardiology, Department of Pediatrics, Children’s of Alabama, University of Alabama Birmingham School of Medicine, Birmingham, AL USA; 10 Division of Cardiology, Department of Pediatrics, University of California San Francisco School of Medicine, San Francisco, CA USA; 11 The Heart Institute, Cincinnati Children’s Hospital Medical Center and Division of Pediatric Cardiology, University of Cincinnati College of Medicine, Cincinnati, OH USA; 12 Division of Pediatric Cardiology, Department of Pediatrics, Stanford University School of Medicine, Stanford, CA USA; 13 Children’s Heart Center Cardiology, Department of Pediatrics, Children’s Healthcare of Atlanta, Emory University School of Medicine, Atlanta, GA USA; 14 The Cardiac Center at the Children’s Hospital of Philadelphia, Philadelphia, PA USA; 15 Connecticut Children’s Hospital, Hartford, CT USA; 16 Pediatric Neurodevelopmental Center, Atlanta, GA USA; 17 Thomas P. Graham Jr. Division of Pediatric Cardiology, Pediatric Heart Institute, Vanderbilt University Medical Center, 2200 Children’s Way, 5230 Doctors’ Office Tower, Nashville, TN 37232 USA

**Correction to: Pediatric Cardiology** 10.1007/s00246-024-03650-2

In the original publication Fig. 2 was incorrectly published; the correct Fig. 2 should have appeared as shown below:

**Fig. 2:**
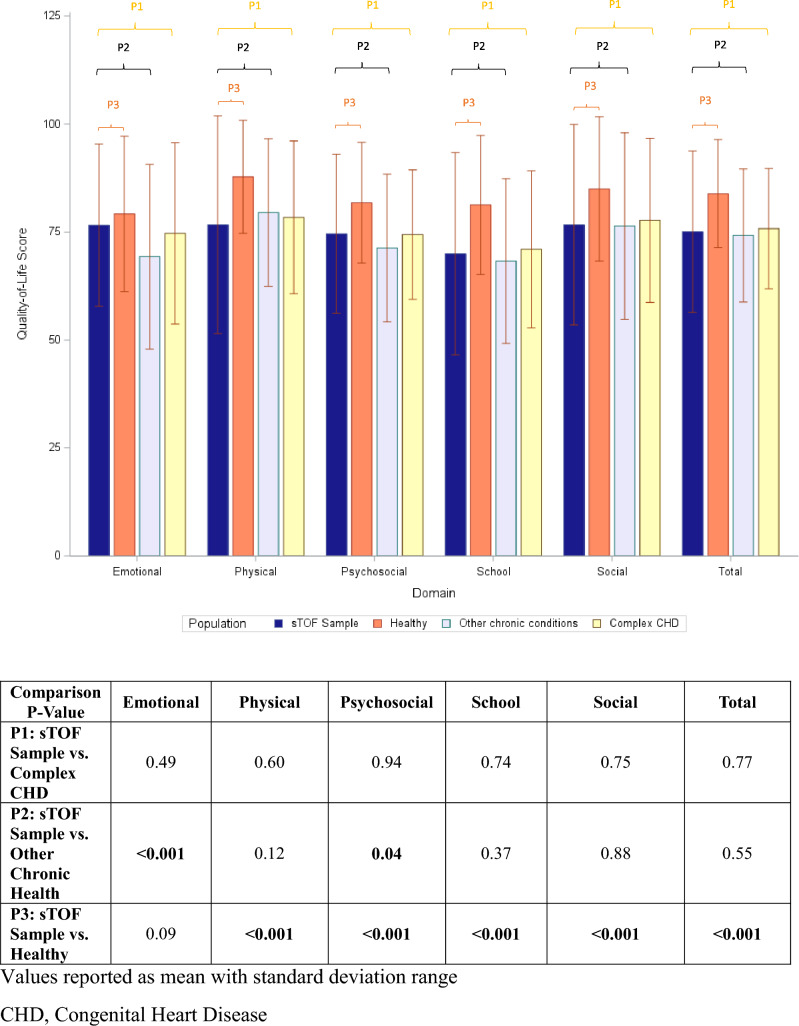
Comparison of quality-of-life scores to healthy children and children with other chronic health conditions

The original article has been corrected.

