# Supplementary material for: Health-Related Quality of Life After Neonatal Treatment of Symptomatic Tetralogy of Fallot: Insights from the Congenital Cardiac Research Collaborative
Source: Pediatr Cardiol. 2024 Sep 21;46(8):2303–14. doi: 10.1007/s00246-024-03650-2 (PMC12583293; doi:10.1007/s00246-024-03650-2)
Supplement: Supplementary file 1 — Supplementary file1 (PDF 93 KB) [file 246_2024_3650_MOESM1_ESM.pdf]

CONGENITAL CATHETERIZATION
RESEARCH COLLABORATIVE

Tetralogy of Fallot: Parental Assessment Survey

Instructions: For each question below, please check the answer(s) that you feel best represent your child with Tetralogy of Fallot (TOF). ***If you prefer not to answer a question**, please leave that question blank.*

Note: By completing this survey, you acknowledge you are the legal guardian.

Today's date (MM/DD/YYYY): ____________________

1. **Indicate your relationship to the child:** [check one]
   - ◻ Mother
   - ◻ Father
   - ◻ Grandparent
   - ◻ Other guardian (specify): ____________________________

2. **Child's date of birth:** (MM/DD/YYYY)
   ___ / ___ / _______

3. **Primary language spoken in your child's home:** [check one]
   - ◻ English
   - ◻ Spanish
   - ◻ Chinese
   - ◻ Other (specify): ______________

4. **Do you need/ require a translator when seeking medical care?** [check one]
   - ◻ Yes
   - ◻ No

5. **Has your child seen a cardiologist in the last 12 months?** [check one]
   - ◻ Yes
   - ◻ No - (If no, reason why your child has not seen a cardiologist in the last 12 months? [check one]
     - ◻ Follow-up not scheduled for the last 12 months
     - ◻ Missed appointment
     - ◻ Do not have a cardiologist
     - ◻ Other (specify): __________________

Please answer Question 6 based on status **at the time your child was born (not current status)**

6. **Highest level of degree child's mother and father received** [check one education level for mother, one for father]

**Education level:**	Mother	Father
Some high school, no degree	◻	◻
High school graduate/ GED or equivalent	◻	◻
Community College/ Vocational school	◻	◻
Some college, no degree	◻	◻
Graduated 4 year college/ University Degree	◻	◻
Professional Degree/ Graduate School	◻	◻
Unknown	◻	◻

Current health status:

Please answer the remaining questions about your child as of today's date

7. **How is your child fed?** [check one]
   - □ All oral (by mouth)
   - □ All tube feedings - If selected, please indicate:
     - □ Nasogastric (NG)
     - □ Nasojejunal (NJ)
     - □ Gastrostomy tube (g-tube)
   - □ Combination of oral and tube feedings

8. **Does your child require a tracheostomy, mechanical ventilation, home oxygen, or any other respiratory support?** [check one]
   - □ No, my child does not require any of these
   - □ Tracheostomy with collar
   - □ Tracheostomy with ventilator
   - □ CPAP (continuous positive airway pressure)
   - □ BiPAP (bi-level positive airway pressure)
   - □ Nasal cannula oxygen
   - □ Other (specify): ____________________

9. **Does your child *currently* receive any of the following services?** [check **all** that apply]
   - □ Occupational therapy
   - □ Physical therapy
   - □ Speech therapy
   - □ Behavioral therapy
   - □ N/A- my child does not receive any of these services

10. **Has your child received or is your child currently receiving any of the following?** [check **all** that apply]
    - □ Any type of early intervention program (for example "Early-On", Head Start, Babies Can't Wait, etc.)
    - □ Individualized Education Program (IEP)/ 504 plan
    - □ Special education services
    - □ None of the above are true for my child

11. **Has your child ever been held back a grade in school due to school performance?** [check one]
    - □ Yes, my child has been held back
    - □ No, my child has never been held back
    - □ N/A- my child is not in school yet

12. **Has your child been diagnosed with any of the following by a medical professional?** [check **all** that apply]
    - □ Developmental delay
    - □ Speech delay
    - □ Hearing loss
    - □ Hearing loss requiring hearing aids
    - □ Vision problems
    - □ Vision problems requiring glasses or contacts
    - □ Problems with motor or movement skills
    - □ Problems with sensory skills/sensory deficit
    - □ Attention Deficit Hyperactivity Disorder (ADHD) or Attention Deficit Disorder (ADD)
    - □ Autism Spectrum Disorder (ASD)
    - □ Learning disability
    - □ None- my child has not been diagnosed with any of the above

13. **Overall, how do you judge your child's health at the present time?** [check one]
    - □ Excellent
    - □ Good
    - □ Fair
    - □ Poor

14. **Overall, how do you judge your child's quality of life at the present time?** [check one]
    - □ Excellent
    - □ Good
    - □ Fair
    - □ Poor

15. **At the present time, do you have any of the following concerns about your child?** [check **all** that apply]
    - □ Growth and nutrition
    - □ School, learning or development
    - □ General health and quality of life
    - □ Social development
    - □ Cardiac concerns
    - □ None
    - □ Other (specify): ____________
